# Early Versus Late Mild Cognitive Impairment: Neural Event-Related Oscillations During a Go/No Go Task

**DOI:** 10.1093/geroni/igab046.437

**Published:** 2021-12-17

**Authors:** Elizabeth Lydon, Lydia Nguyen, Shraddha Shende, Hsueh-Sheng Chiang, Raksha Mudar

**Affiliations:** 1 University of Illinois Urbana-Champaign, Champaign, Illinois, United States; 2 iN, 2L, Denver, Colorado, United States; 3 UT Southwestern Medical Center, Texas, United States; 4 University of Illinois-Urbana Champaign, Champaign, Illinois, United States

## Abstract

Amnestic mild cognitive impairment (aMCI) is marked by episodic memory deficits, which is used to classify individuals into early MCI (EMCI) and late MCI (LMCI). Growing evidence suggests that individuals with EMCI and LMCI differ in other cognitive functions including cognitive control, but these are less frequently studied. Using a semantic Go/NoGo task, we examined differences in cognitive control between EMCI and LMCI on behavioral (accuracy and reaction time) and neural (scalp-recorded event-related oscillations in theta and alpha band) measures. Although no behavioral differences were observed between the groups, EMCI and LMCI groups differed in patterns of neural oscillations for Go compared to NoGo trials. The EMCI group showed differences in theta power at central electrodes and alpha power at central and centro-parietal electrodes between Go and NoGo trials, while the LMCI group did not exhibit such differences. Furthermore, the LMCI group had higher theta synchronization on Go trials at central electrodes compared to the EMCI group. These findings suggest that while behavioral differences may not be observable, neural changes underlying cognitive control processes may differentiate EMCI and LMCI stages and may be useful to understand the trajectory of aMCI.

